# Psychometric properties and minimal important differences of SF-36 in Idiopathic Pulmonary Fibrosis

**DOI:** 10.1186/s12931-019-1010-5

**Published:** 2019-03-01

**Authors:** Sabine Witt, Ekaterina Krauss, María Asunción Nieto Barbero, Veronika Müller, Philippe Bonniaud, Carlo Vancheri, Athol U. Wells, Martina Vasakova, Alberto Pesci, Walter Klepetko, Werner Seeger, Bruno Crestani, Reiner Leidl, Rolf Holle, Larissa Schwarzkopf, Andreas Guenther

**Affiliations:** 1 Helmholtz Zentrum München, German Research Center for Environmental Health (GmbH), Institute of Health Economics and Health Care Management, Member of the German Center for Lung Research (DZL), Comprehensive Pneumology Center Munich (CPC-M), Neuherberg, Germany; 2 European IPF Registry & Biobank (eurIPFreg/bank), Giessen, Germany; 3 Universities of Giessen and Marburg Lung Center (UGMLC), Member of the German Center for Lung Research (DZL), Giessen, Marburg, Germany; 4 Hospital Clinico San Carlos, Madrid, Spain; 5 Department of Pulmonology, Semmelweis University, Budapest, Hungary; 6 Reference Center for Rare Pulmonary Diseases, Centre Hospitalier Universitaire Dijon-Bourgogne, INSERMU1231, Université Bourgogne/Franche-Comté, Dijon, France; 7 Department of Clinical and Molecular Biomedicine, Università degli Studi di Catania, Catania, Italy; 8 Interstitial Lung Disease Unit, Royal Brompton Hospital, London, UK; 9 First Faculty of Medicine, Thomayer University Hospital Prague, Prague, Czech Republic; 10 Ospedale San Gerardo, Monza, Italy; 11 Department of Thoracic Surgery, Vienna University Hospital, Vienna, Austria; 12 Cardio-Pulmonary Institute (CPI), EXC 2026, Project ID: 390649896, Justus-Liebig University Giessen , Giessen, Germany; 13 Institut National de la Santé et de la Recherche Médicale (INSERM 700), Hôpital Bichat, Service de Pneumologie, Paris, France; 14 Ludwig-Maximilians-University, Munich Center of Health Sciences, Munich, Germany; 15 AGAPLESION Lung Clinic Waldhof-Elgershausen, Greifenstein, Germany

**Keywords:** Idiopathic pulmonary fibrosis, Rare diseases, Quality of life, Patient-reported outcome

## Abstract

**Background:**

Idiopathic pulmonary fibrosis (IPF) is a rare disease with a median survival of 3–5 years after diagnosis with limited treatment options. The aim of this study is to assess the psychometric characteristics of the Short Form 36 Health Status Questionnaire (SF-36) in IPF and to provide disease specific minimally important differences (MID).

**Methods:**

Data source was the European IPF Registry (eurIPFreg). The psychometric properties of the SF-36 version 2 were evaluated based on objective clinical measures as well as subjective perception. We analysed acceptance, feasibility, discrimination ability, construct and criterion validity, responsiveness and test-retest-reliability. MIDs were estimated via distribution and anchor-based approaches.

**Results:**

The study population included 258 individuals (73.3% male; mean age 67.3 years, SD 10.7). Of them 75.2% (194 individuals) had no missing item. The distribution of several items was skewed, although floor effect was acceptable. Physical component score (PCS) correlated significantly and moderately with several anchors, whereas the correlations of mental component score (MCS) and anchors were only small. The tests showed mainly significant lower HRQL in individuals with long-term oxygen therapy. Analyses in stable individuals did not show significant changes of HRQL except for one dimension and anchor. Individuals with relevant changes of the health status based on the anchors had significant changes in all SF-36 dimensions and summary scales except for the dimension PAIN. PCS and MCS had mean MIDs of five and six, respectively. Mean MIDs of the dimensions ranged from seven to 21.

**Conclusion:**

It seems that the SF-36 is a valid instrument to measure HRQL in IPF and so can be used in RCTs or individual monitoring of disease. Nevertheless, the additional evaluation of longitudinal aspects and MIDs can be recommended to further analyse these factors. Our findings have a great potential impact on the evaluation of IPF patients.

**Trial registration:**

The eurIPFreg and eurIPFbank are listed in https://clinicaltrials.gov (NCT02951416).

**Electronic supplementary material:**

The online version of this article (10.1186/s12931-019-1010-5) contains supplementary material, which is available to authorized users.

## Background

Idiopathic pulmonary fibrosis (IPF) is a rare disease with a median survival of 3–5 years after diagnosis [1]. Current treatment options as pirfenidone and nintedanib are still limited in respect to prolonging life [2]. Mortality alone does not appear to be a sufficient clinical endpoint regarding patients’ outcomes [1, 3–5]. Thus, health-related quality of life (HRQL) as a patient-reported outcome gains relevance [6]. Existing HRQOL instruments are not yet sufficiently validated as clinically meaningful endpoints in IPF [7–9]. Therefore, the utilisation of validated HRQL instruments is strongly recommended for marketing-authorisation application of novel treatments [10, 11].

The Short Form 36 Health Status Questionnaire (SF-36) is a generic instrument [12] which is frequently used in clinical trials in IPF as a secondary endpoint [13–15]. Generic HRQOL instruments are designed to measure overall health states and allow comparisons across patients with different diseases and the general population. Evaluating the validity of these generic instruments in specific diseases is indispensable and is also needed for the SF-36 in IPF [9]. Currently, two studies provide psychometric characteristics of the SF-36 in IPF based on longitudinal data [16, 17]. It is our knowledge that only these studies analysed if the SF-36 can detect changes or stability over time of HRQL, which is essential as an endpoint in clinical trials. Tomioka et al. used observational data of a single outpatient centre in Japan [16]. The analysis of Swigris et al. was based on international multicentre-data, which were part of the randomised clinical trial BUILD-1. Thus, the study population was subject to numerous inclusion and exclusion criteria [17, 18]. Hence, the external validity of the results of both studies might be reduced. Belkin et al. proposed additional research should take place before a broad implementation of the SF-36 [8]. Moreover, only Swigris et al. provide disease specific minimally important differences (MID), which are obligatory to evaluate changes in QOL over time [17, 19]. Therefore, patients would benefit from further longitudinal analysis based on multicentre-data and in a real-world setting.

The aim of this study was (1) to assess the psychometric characteristics of the SF-36 in IPF (acceptance and feasibility; discrimination ability; construct and criterion validity, and internal consistency; responsiveness and test-retest- reliability). Furthermore, we intended (2) to evaluate disease specific MIDs, using data from a comprehensive European registry, which provides real-world data from patients in different disease stages and ethnical backgrounds.

## Materials and methods

### Data and participants

Data source was the European IPF Registry (eurIPFreg), one of Europe’s leading IPF longitudinal databases with nine participating countries and eleven study centres [20]. Both, eurIPFreg and eurIPFbank (biobank of eurIPFreg) have been reviewed and received positive votes from institutional review boards in Germany (e.g. Ethics Committee of Justus-Liebig-University of Giessen; 111/08), France, Italy, Austria, Spain, Czech Republic, Hungary and the UK. The research was conducted strictly according to the principles of the Declaration of Helsinki. The eurIPFreg and eurIPFbank are listed in ClinicalTrials.gov (NCT02951416). Patients were included into the registry starting November 2009. The datasets generated and investigated during the current study are not publicly available due to registry regulations, but are available from the corresponding author on reasonable request and agreement of the Principle Investigators of the eurIPFreg.

Patients’ data were collected by standardised questionnaires for physicians and patients at baseline and follow-up visits with intervals of three to six months, considering individual necessity and practical issues. Interim documentation in case of unscheduled visits was possible. The collected data was comprehensive and included besides clinical measurements and demographic data, also patient-self-reported instruments [21].

The study population was comprised of incident and prevalent IPF patients. There were following exclusion criteria: subjects without information of sex and age, absence of IPF diagnosis validated by a multidisciplinary team, missing lung function test at baseline, absent or incomplete information on SF-36 items (more than 50% missing values within each dimension) [22]. In case of missing date of filling out the questionnaires or medical examinations, we used the predefined follow-up date.

### HRQL instrument

The SF-36 version 2 was used [22]. It contains 36 items categorised into 8 dimensions (vitality (VITAL), physical functioning (PFI), bodily pain (PAIN), general health perceptions (GHP), physical role functioning (ROLPH), emotional role functioning (ROLEM), social role functioning (SOCIAL), mental health (MHI)) and a physical as well as a mental component score (PCS and MCS), which can be calculated for individuals providing all dimensions. The dimensions range from zero to 100; higher values imply higher functional health and well-being. The PCS and MCS are adjusted to normal distribution (mean equal 50, standard deviation (SD) equal 10) with higher values for better functional health and well-being. Scores were calculated based on German scoring system to provide comparability since the majority of considered patients were Germans [23].

### Anchors

For purposes of examining the validity of the SF-36 in IPF, we used the following anchors at baseline and follow-up: 6 min walking distance (6MWD) [24–26], percent of the predicted value of forced vital capacity (FVC % pred) (based on Global Lungs Initiative (GLI) equations), percent of predicted value of carbon monoxide diffusion capacity of the lung (corrected for haemoglobin, and if not available uncorrected values (DLCO % pred)), and also modified New York Heart Association Classification (NYHA) grade, evaluated by the physician (I-IV, the higher the more impaired) [27],

Baseline Dyspnoea Index (BDI) (scale 0–12, the lower the more impaired) (baseline only) and Transitional Dyspnoea Index (TDI) (scale − 9 to 9, the lower the more impaired) (follow-up only) [28], long-term oxygen therapy (LTOT) (baseline only), Modified Medical Research Council (mMRC) Dyspnea Scale (1–5, the higher the more impaired) (baseline only) [29], and an item of the SF-36 which indicates perceived change in health during the previous year (follow-ups only). This SF-36 item was not included in any of the dimensions and component scores [12, 22].

### Cross-sectional analysis

The SF-36 value was not captured during the first visit in all cases. Therefore, in this study we defined baseline as the date of the first filled in SF-36. Additionally, not all examinations were performed at each visit and we therefore decided to accept anchors within a timeframe of plus/minus 45 days around the first visit filled in SF-36. The time frame of 45 days was chosen because frequently, the date was only given as month/year and we needed to set the day to the 15th. Since the SF-36 considers the health status of the last 4 weeks and in some cases the exact date of examination was set to the mid of month, we decided to use 45 days as the maximum interval between anchors and SF-36.

### Acceptance and feasibility

To assess acceptance and feasibility we examined the frequency of missing responses to items. As there might be some differences in specific populations, we searched for a possible influence of age, gender and severity of disease (estimated by DLCO % pred, FVC % pred, 6MWD) on the frequency of missing items via Pearson and Spearman correlation for metric and categorical variables, respectively.

### Discrimination ability

Ceiling and floor effects in single items were examined as a possible indicator of an insufficient discrimination ability.

### Construct and criterion validity, and internal consistency

The construct validity of the domains and summary measures was checked for individuals with and without LTOT via Wilcoxon-Mann-Whitney test to consider possible non-normal distribution. We assumed that individuals with LTOT have a lower HRQOL than individuals without [30].

The criterion validity of the domains and summary measures was evaluated via Pearson correlation in case of metric anchors and Spearman correlation in case of ordinal anchors. A better health status and thus better values of the anchors should implicate higher HRQL and vice versa. Strength of correlation was categorized according to Cohen in great (greater than 0.5), moderate (0.3–0.5), small (0.1–0.3), and trivial (less than 0.1) [31]. Internal consistency was assessed with Cronbach’s alpha for the domains and summary scores of the SF-36.

### Longitudinal analysis

Considering the flexible intervals between the visits, the time frame between baseline and follow-up could not be defined a priori. As the SF-36 evaluates the HRQOL of the last four weeks, the interval between baseline and follow up needed to be of at least 28 days, except the SF-36 change item which has a time horizon of one year, here we considered only follow-ups with an interval of 300 to 450 days.

Consistent with the baseline procedure, the follow-up anchors were selected within a time frame of plus/minus 45 days around a filled in SF-36 form. For this purpose, we used a stepwise approach to find the nearest anchor around the SF-36 measurement and excluded matched anchors before we started the next search. An anchor examination was never used for two SF-36 measurements. The number of follow up visits with documented HRQOL and anchors varied and could possibly be more than one. In order to improve the power of these analyses, we decided to use the first and last observation per anchor and individual, provided their health status (improved vs. baseline, deteriorated vs. baseline, same as baseline) varied between these two observations. For example, if the health status was initially stable but deteriorated afterwards, we used both events in different groups and therefore different analyses. Considering an individual twice in one group (e.g. deterioration) would have lead to a bias. In this case, we considered only the last measurement of the respective anchor. For TDI we used only one observation, which was plus/minus 45 days around a filledin SF-36 compared to the preceding SF-36 as the instrument measures the change between two visits.

### Responsiveness and test-retest- reliability

For assessing responsiveness and test-retest-reliability the individuals were categorized depending on whether their health status and thus their anchors changed during the follow-up or not. We defined variations with more than the MID of the anchor as improvement and deterioration, respectively. If the shift from baseline to follow up was less than the MID, we defined the anchor as unchanged. We defined the following MIDs for the changes of the anchors: 6MWD ≥30 m [32–34], FVC % pred ≥10%, and DLCO % pred ≥15% [35], TDI =1 [28, 36], modified NYHA score ≥ 1 [37]. If the anchor is stable, there should not be a significant difference in the SF-36 between baseline and follow up (test-retest-reliability). The responsiveness was tested by comparing baseline and follow up values of the SF-36 for improved and deteriorated anchors separately. A relevant change of the anchors should implicate a significant shift of HRQL. We used Wilcoxon signed-rank test in case to consider possible non-normal distribution of differences and possible small numbers of observations within the anchors per group.

### Minimal important difference (MID)

The MIDs of the summary scores and the dimensions were estimated anchor- and distribution-based. To obtain distribution-based MIDs we used half standard deviation (SD) of baseline values of normally distributed domains [38, 39]. Normality was evaluated by visual inspection [38, 39].

For anchor-based MIDs, only anchors providing a correlation ≥0.3 at baseline to ensure sufficient relationships were considered [31, 39]. MIDs were estimated via linking, which are unaffected by the degree of correlation [40]. Therefore, the MID of the anchor was multiplied by the quotient of the baseline SD of the HRQL domain and the baseline SD of the anchor.

$$ {MID}_{HRQL}={MID}_{anchor}\times \left({SD}_{HRQL}/{SD}_{anchor}\right) $$

As only metric anchor provide meaningful SD, categorical anchors needed to be excluded and only following metric anchors were used: 6MWD, FVC % pred, and DLCO % pred.The mean of distribution- and anchor-based MIDs (if normally distributed and anchor correlated significantly and r ≥ 0.3) was calculated to provide an overall estimate of the specific MID. Additionally, the mean of the distribution-based MID with the MID of the anchor with the highest correlation was provided.

#### Sensitivity analysis

To detect possible bias we tested a possible influence of study sites on HRQL, adjusted for age, gender, DLCO % pred, FVC % pred and 6MWD.

All statistical analyses were performed using SAS software (version 9.3,©2002–2010 by SAS Institute Inc., Cary, NC, USA).

## Results

### Cross-sectional analysis

Out of 528 IPF patients, we excluded 139 patients as they had no SF-36 and one individual who had only answered one question. From the resulting 388 patients we excluded three individuals without information on gender and six individuals without date of birth. From the remaining 379 individuals, there was no FVC measurement around the first SF-36 in 121 cases. That does not mean there was no FVC measurement at all, but not within 45 days around the first SF-36. The study population included 258 individuals (73.3% male) with a mean age of 67.3 years (SD 10.7) and on average 2.6 years since first diagnosis (SD 2.8). In spite of a tolerance, a period of plus/minus 45 days between SF-36 and anchor, it was not possible to provide all anchors for each patient. HRQL presented in MCS and PCS was considerably reduced compared with norm values (mean 45.3, SD 11.8 and mean 34.6, SD 10.5 versus mean 50.0, SD 10.0) (Table 1). Except for ROLEM and ROLPH all HRQL measures were normally distributed based on visual validation.

**Table 1:** Baseline characteristics

		n valid	n missing	mean / frequency	sd / %
Age (in years)	mean (sd)	258	0	67.3	10.7
Gender	frequency (%)	258	0		
	male			189	73.3
Study site	frequency (%)	258	0		
	Paris			73	28.3
	Giessen			153	59.3
	London			9	3.5
	Vienna			12	4.7
	Prague			3	1.2
	Napoli			1	0.4
	Budapest			6	2.3
Smoker	frequency (%)				
	current or ever	248	10	181	73.0
Time since diagnosis (in years)	mean (sd)	249	9	2.6	2.8
FVC (in percent of predicted)	mean (sd)	258	0	62.7	19.8
DLCO (in percent of predicted)	mean (sd)	228	30	41.8	18.8
6MWD (in meters)	mean (sd)	181	77	403.1	120.1
LTOT	frequency (%)				
	current	244	14	82	33.6
LTx	frequency (%)	239	19		
	listed			3	1.3
	transplantated			4	1.7
SF-36	mean (sd)				
	PCS	232	26	34.6	10.5
	MCS	232	26	45.3	11.8
	GHP	250	8	40.3	18.4
	PFI	253	5	42.2	27.8
	ROLPH	247	11	30.2	40.5
	ROLEM	244	14	56.2	45.8
	SOCIAL	257	1	62.0	28.5
	MHI	250	8	62.5	21.3
	PAIN	255	3	65.1	28.2
	VITAL	253	5	40.0	18.9
BDI (range 0–12)	frequency (%)	140	118		
	4			2	1.4
	5			3	2.1
	6			44	31.4
	7			48	34.3
	8			30	21.4
	9			10	7.1
	10			3	2.1
mMRC (range 1–5)	frequency (%)	196	62		
	1			35	17.9
	2			61	31.1
	3			44	22.5
	4			27	13.8
	5			29	14.8
Modified NYHA scale (1–4)	frequency (%)	229	29		
	1			23	10.0
	2			97	42.4
	3			95	41.5
	4			14	6.1

Abbreviations: *sd* standard deviation, *FVC* forced vital capacity, *DLCO* Diffusing capacity of the lungs for carbon monoxide, *6MWD* 6 min walking distance, *LTOT* Long-term oxygen therapy, *LTx* Lung transplantation, *SF-36, BDI* Baseline Dyspnoea Index, *mMRC* Modified Medical Research Council Dyspnea Scale, *NYHA* New York Heart Association

### Acceptance and feasibility

Regarding single items, 75.2% (194 individuals) had no missing item in the SF-36, 21.3% (*n* = 55) one to ten and 3.5% (*n* = 9) eleven to 28 missing items. The number of missing items and age (r = 0.13, *p* = 0.03) correlated significantly. Gender as well as severity of disease were of no significant influence. A graphic representation on item level can be found in the Additional file 1 Figure S1. Within the dimensions, the percentage of completely answered items ranged from 93.0% (ROLEM) to 95.7% (PAIN) (Table 2).

**Table 2:** Missing items within the dimensions

		GHP		PFI		ROLPH		ROLEM		SOCIAL		MHI		PAIN		VITAL	
Number of items within dimension		5		10		4		3		2		5		2		4	
Number of missing values (%)	0	242	93.8	234	90.7	241	93.41	240	93.02	245	94.96	244	94.57	247	95.74	246	95.35
	1	6	2.33	12	4.65	5	1.94	4	1.55	12	4.65	5	1.94	8	3.1	4	1.55
	2	2	0.78	4	1.55	1	0.39	5	1.94	1	0.39	1	0.39	3	1.16	3	1.16
	3	2	0.78	1	0.39	8	3.1	9	3.49			2	0.78				
	4	6	2.33			3	1.16					2	0.78			5	1.94
	5			2	0.78							4	1.55				
	6																
	7			1	0.39												
	8			2	0.78												
	9			1	0.39												
	10			1	0.39												

Abbreviations: *GHP* General health perceptions, *PFI* Physical functioning, *ROLPH* Physical role functioning, *ROLEM* emotional role functioning, *SOCIAL* Social role functioning, *MHI* Mental health, *PAIN* bodily pain, *VITAL* Vitality

### Discrimination ability

The distributions of several items were skewed, six had a tendency of more than 60% towards the worst answer category: ROLPH 1–4 (67.9, 74.3, 69.1 and 69.1%) and PFI 1 (78.9%) and 4 (65.6%). Almost half of the study population rejected (answer: ‘definitely false’) that their ‘health is excellent’ (45.8%, item 5 of GHP, possible answers: definitely true; mostly true; don’t know; mostly false; definitely false) (Additional file 2 Figure S2).

### Construct and criterion validity, and internal consistency

PCS correlated significantly and moderately with several anchors whereas MCS did not correlate with any anchor with r ≥ 0.3. ROLEM, MHI and PAIN did not reach moderate or high correlations either. Other dimensions correlated significantly with particular anchors on a moderate to high level (Table 3). The tests showed significant lower HRQL in individuals with LTOT except for MCS, MHI, and PAIN (Table 4). Cronbach’s alpha ranged from 0.85 (SOCIAL) to 0.87 (ROLEM), MCS and PCS showed a good internal consistency as well (0.86 both).

**Table 3:** Criterion validity analysed via correlation coefficiants

	n^c^	PCS	MCS	PFI	ROLEM	ROLPH	GHP	MHI	VITAL	SOCIAL	PAIN
FVC % pred^a^	232–257	0.35**	− 0.01	0.35**	0.11	0.35**	0.26**	< 0.01	0.20*	0.11	0.07
DLCO % pred^a^	208–227	0.36**	0.05	0.39**	0.14*	0.37**	0.22*	0.04	0.20*	0.23*	0.02
6MWD^a^	167-180	0.44**	0.17*	0.53**	0.18*	0.37**	0.29*	0.22*	0.32**	0.31**	0.17*
mMRC^b^	180-196	−0.48**	− 0.11	−0.61**	− 0.14	−0.3**	− 0.32**	− 0.13	−0.37**	− 0.31**	− 0.16*
BDI^b^	131-140	0.25*	− 0.05	0.38**	− 0.02	0.11	0.09	0.01	0.22*	0.14	0.14
NYHA^b^	206–228	−0.33**	− 0.09	−0.41**	− 0.17*	− 0.37**	− 0.22*	− 0.13*	− 0.3**	− 0.18*	− 0.09

^a^Pearson correlation

^b^Spearman correlation

^c^sample size varying depending on temporal relation of anchors and filled in SF-36, number of missing items within SF-36 and the possibility to calculate dimensions and summary scores

*p < 0.05

**p < 0.0001

bold at least moderate criterion validity (r ≥ 0.3)

Abbreviations: *FVC* pred forced vital capacity percent predicted, *DLCO* pred diffusing capacity of the lungs for carbon monoxide percent predicted, *6MWD* 6 min walking distance, *mMRC* Modified Medical Research Council Dyspnea Scale, *BDI* Baseline Dyspnoea Index, *NYHA* New York Heart Association, *GHP* general health perceptions, *PFI* physical functioning, *ROLPH* physical role functioning, *ROLEM* emotional role functioning, *SOCIAL* social role functioning, *MHI* Mental health, *PAIN* bodily pain, *VITAL* Vitality

**Table 4:** Construct validity: mean difference of QOL between patients without and with long-term oxygen therapy; significant differences of QOL confirm criterion validity

n^a^ = 220–243	mean difference	sd
PSC	7.7**	9.9
MSC	4.0	11.9
PFI	25.4**	25.7
ROLEM	19.9*	45.3
ROLPH	25.7**	38.8
GHP	10.2*	18.9
MHI	5.9	21.2
VITAL	10.4*	19.4
SOCIAL	19.7*	28.2
PAIN	2.4	28.3

a sample size varying depending on temporal relation of anchors and filled in SF-36, number of missing items within SF-36 and the possibility to calculate dimensions and summary scores

*p < 0.05

**p < 0.001

Abbreviations: *QOL* Quality of life, *sd* standard deviation, *PCS* Physical component score, *MCS* Mental component score, *GHP* General health perceptions, *PFI* Physical functioning, *ROLPH* physical role functioning, *ROLEM* emotional role functioning, *SOCIAL* Social role functioning, *MHI* Mental health, *PAIN* bodily pain, *VITAL* vitality

### Longitudinal analysis

SF-36 follow-up data were available of 161 individuals, where almost half of them (78, 48.5%) had up to four further documentations of HRQL and the maximum of filled in SF-36 was 10. The mean time between baseline and all considered follow-ups was 1.3 years (SD 0.88, range 0.1–5.0 years). The number of considered matches of anchors and HRQL (*n* = 591) was higher than the number of individuals within the follow-up study population, as different visits per patient needed to be considered to provide as much timely congruent documented anchors and filled in SF-36 questionnaires per individual as possible. Moreover, we accepted individuals twice with their first and last observation per anchor, if their health status of the respective anchor varied.

### Test-retest-reliability and responsiveness

Analyses for test-retest-reliability did not show significant differences of HRQL except for SOCIAL and the anchor FVC % pred (Table 5). Individuals with relevant changes of the health status based on the anchors had significant changes in all SF-36 dimensions and summary scales except for PAIN (responsiveness) (Table 6).

**Table 5:** Test-retest-reliability: mean change of QOL in stable health status in anchor; non-significant changes of QOL confirm test-retest-reliability

	FVC		DLCO		6MWD		SF-36 item		TDI		NYHA	
n^a^	82–106		73–89		31–33		28–28		22–24		55–67	
	mean	sd	mean	sd	mean	sd	mean	sd	mean	sd	mean	sd
PSC	−0.7	8.4	−0.4	7.7	−0.8	7.0	0.6	9.4	−0.3	7.9	−0.7	8.3
MSC	−0.7	10.0	−0.2	9.9	−1.0	8.7	−0.3	7.0	0.1	8.7	−0.7	10.5
PFI	−1.0	19.7	−1.7	20.7	−4.7	18.1	2.8	21.9	1.6	21.4	−3.0	21.3
ROLEM	−0.7	51.9	−0.9	47.8	−2.1	43.9	0.0	46.3	12.1	43.1	−3.3	51.9
ROLPH	−1.3	35.9	−1.2	32.8	−5.3	31.7	6.8	42.1	6.1	35.0	0.8	35.9
GHP	−1.3	16.8	−0.5	17.0	−5.0	19.1	−1.3	15.7	−0.9	12.9	−0.1	18.5
MHI	−1.7	15.6	−0.9	15.8	−4.3	15.2	0.2	14.0	−0.3	13.9	−0.7	15.9
VITAL	−0.1	16.1	−1.2	16.2	−1.8	14.1	1.0	13.7	−7.2	17.6	−1.5	14.8
SOCIAL	−3.4*	21.4	−1.0	22.9	0.0	21.7	−5.3	17.1	−1.6	25.1	−4.3	23.5
PAIN	0.2	28.3	1.2	27.3	2.5	24.3	0.4	29.2	−6.2	26.4	−2.2	28.3

a sample size varying depending on temporal relation of anchors and filled in SF-36, number of missing items within SF-36 and the possibility to calculate dimensions and summary scores

*p < 0.05

**p < 0.001

Abbreviations: PCS physical component score, MCS mental component score, GHP general health perceptions, PFI physical functioning, ROLPH physical role functioning, ROLEM emotional role functioning, SOCIAL social role functioning, MHI mental health, PAIN bodily pain, VITAL vitality, sd standard deviation, FVC forced vital capacity, DLCO diffusing capacity of the lungs for carbon monoxide, 6MWD 6 min walking distance, SF-36 item indicating health status of the last year, TDI Transitional Dyspnoea Index, NYHA New York Heart Association

**Table 6:** Responsiveness: mean change of QOL in changed health status in anchor; significant changes of QOL confirm responsiveness

Improvement in the specific anchor												
	FVC		DLCO		6MWD		SF-36 item		TDI		NYHA	
n^a^	9–11		10–11		14–17		10–15		15–16		18–19	
	mean	sd	mean	sd	mean	sd	mean	sd	mean	sd	mean	sd
PSC	6.2*	6.5	−3.9	9.6	3.0	10.5	4.0	10.1	4.3*	11.1	1.8	9.4
MSC	−3.6	13.1	−1.8	10.5	−1.1	10.2	−0.7	9.0	−0.7	8.3	−1.4	7.3
PFI	15.3	24.0	−3.9	19.9	1.6	19.7	5.4*	11.7	5.5	18.9	−3.2	24.1
ROLEM	−16.7	72.4	0.0	64.8	−8.9	47.9	0.0	62.9	−6.3	42.5	−20.4*	36.4
ROLPH	17.5	39.2	−25.0	45.6	11.7	28.1	30.0	55.0	20.3	44.0	2.8	34.2
GHP	7.1	20.8	−11.9*	14.6	2.3	14.5	4.1	24.2	5.0	22.8	2.6	19.4
MHI	2.0	19.0	−4.2	10.2	3.2	11.7	−1.0	9.6	1.9	16.7	1.7	11.7
VITAL	4.5	12.8	−2.3	14.0	4.4	13.4	4.0	16.5	7.4	14.8	1.1	14.1
SOCIAL	1.1	30.8	−10.2*	9.4	0.0	15.3	1.8	18.9	3.1	23.0	6.6	21.0
PAIN	0.2	31.9	4.9	23.9	11.5	28.8	1.3	23.8	−0.3	36.1	6.9	29.6
deterioration in the specific anchor												
	FVC		DLCO		6MWD		SF-36 item		TDI		NYHA	
n^a^	30–36		22–28		29–36		37–45		55–73		29–40	
	mean	sd	mean	sd	mean	sd	mean	sd	mean	sd	mean	sd
PSC	−2.6	10.3	−5.0	10.4	−2.8*	8.5	−3.0*	9.5	−2.4	7.8	−1.1	9.0
MSC	−2.9	13.0	−2.8	10.0	0.1	9.6	−3.0*	9.5	−0.7	8.3	−1.0	11.0
PFI	−12.9*	23.8	−14.1*	25.0	−9.3*	21.4	−10.2*	22.3	−3.9	21.3	− 2.7	25.2
ROLEM	−7.3	62.1	−15.9	49.1	−3.1	44.3	−4.4	48.5	−1.7	37.9	2.2	57.7
ROLPH	−2.3	51.7	−15.0	36.8	−14.5*	33.0	−10.1	35.8	−9.0*	35.7	−4.9	39.6
GHP	−7.3*	17.1	−6.9	16.8	−2.3	17.4	−5.0	15.0	−4.8*	13.8	−6.7	17.1
MHI	−8.1	17.2	−4.4	17.3	−2.1	14.5	−5.8	17.3	−4.1*	13.5	−3.3	17.7
VITAL	−8.2*	18.8	−3.7	22.0	−3.0	15.7	−6.8*	15.0	−4.7*	15.8	−0.4	19.3
SOCIAL	−10.8*	29.6	−12.9*	25.6	−7.3	30.5	− 13.3*	24.8	−1.5	23.2	− 14.1*	32.2
PAIN	−3.1	32.6	−10.0	35.1	4.6	27.4	−3.3	37.9	−2.7	27.3	−8.1	31.0

a sample size varying depending on temporal relation of anchors and filled in SF-36, number of missing items within SF-36 and the possibility to calculate dimensions and summary scores

SD standard deviation

*p < 0.05

**p < 0.001

Abbreviations: *PCS* Physical component score, *MCS* Mental component score, *GHP* General health perceptions, *PFI* Physical functioning, *ROLPH* physical role functioning, *ROLEM* emotional role functioning, *SOCIAL* Social role functioning, *MHI* Mental health, *PAIN* bodily pain, *VITAL* vitality, sd standard deviation, *FVC* Forced vital capacity, *DLCO* Diffusing capacity of the lungs for carbon monoxide, *6MWD* 6 min walking distance, *SF-36* item indicating health status of the last year, *TDI* Transitional Dyspnoea Index, *NYHA* New York Heart Association

### Minimal important difference (MID)

The normal distribution could not be assumed for ROLEM and ROLPH and valid distribution-based MIDs could not be provided for both dimensions. As we considered only anchors with a correlation of at least 0.3 and none of the anchors correlated sufficiently with MCS, ROLEM, GHP, MHI and PAIN, it was not possible to provide any anchor based-MIDs for them. Combining the criteria of normal distribution and an at least moderate correlation, it was not possible to calculate a MID for ROLEM. The overall mean MID of PCS and MCS were five and six, respectively. Mean MIDs of the dimensions ranged from seven to 21 based on anchors correlating with r ≥ 0.3 and estimated MIDs of normally distributed domains and summary scores. Taking only distribution-based values and the MID of the anchor with the highest correlation, the mean MIDs ranged from seven to 14 (Table 7).

**Table 7:** Minimal important differences (MID)

		PCS	MCS	PFI	ROLEM	ROLPH	GHP	MHI	VITAL	SOCIAL	PAIN
Distribution based											
	1/2 sd	5.2	5.9	13.9			9.2	10.6	9.4	14.3	14.1
Anchor based via linking											
	FVC	5.3		14.1		20.5					
	DLCO	8.4		22.2		32.3					
	6MWD	2.6		6.9		10.1			4.7	7.1	
MID	Range^a^	2.6–8.4	5.9	10.1–22.2		10.1–32.3	9.2	10.6	4.7–9.4	7.1–14.3	14.1
	mean all^a^	5	6	14		21	9	11	7	11	14
	mean selected^b^	4	6	10		10	9	11	7	11	14

^a^ based on anchor (if estimate is significant, *r* ≥ 0.3 or R^2^ ≥ 0.09) and distribution based MID (in case of normal distribution)

^b^ based on anchor with the highest correlation (if estimate is significant, *r* ≥ 0.3 or R2 ≥ 0.09) and distribution based MID (in case of normal distribution)

Abbreviations: *sd* standard error, *SEM* Standard error of the mean, *FVC* pred forced vital capacity percent predicted, *DLCO* pred diffusing capacity of the lungs for carbon monoxide percent predicted, 6MWD 6 min walking distance, *PCS* Physical component score, *MCS* Mental component score, *GHP* General health perceptions, *PFI* Physical functioning, *ROLPH* physical role functioning, *ROLEM* emotional role functioning, *SOCIAL* Social role functioning, *MHI* Mental health, *PAIN* bodily pain, *VITAL* vitality

Sensitivity analysis.

The patients of the study sites varied in HRQL, disease severity, age and gender. After adjusting for age, gender, DLCO % pred, FVC % pred and 6MWD there was no influence of study site on HRQL detectable.

## Discussion

The SF-36 seems to provide adequate psychometric properties to assess HRQL in IPF cohort. Our analysis demonstrated an increased number of missing items in older patients [41]. It is well known, that in an older population the number of missing items is higher [42, 43]. Especially items containing the wording ‘work or other regular daily activity’ (dimensions ROLEM and ROLPH) led to a higher number of missing values in our study as well as in the studies of Hayes et al. and Mallinson [42, 43].

A possible reason could be a misunderstanding of the wording ‘work or other regular daily activity’ as probably most of the older participants were retired or not able to hold down a regular job [42]. As 75.2% of participants completed the questionnaire without any missing values in our study, we assumed that the higher age of most of the patients suffering IPF is not necessarily a limiting factor.

As we expected in a severe disease such as IPF, there was a floor effect of the items regarding limitations in ‘vigorous activities’ and ‘climbing several flights of stairs’ (dimension PFI) as well as the statement ‘my health is excellent’ (dimension GHP). As the dimension PFI contains ten items and considers different levels of activities, the floor effect of two items may be acceptable. Surprisingly, 4.4 and 7.9% of our study population declared to have no limitations at all in these two physical activity categories and 1.6% rated their health as excellent.

Construct validity was also given. However, the measured dimensions MHI and PAIN and the MCS were not significantly reduced in individuals suffering LTOT. This might be caused by a positive influence of LTOT on well-being in some IPF patients. Regarding the criterion validity, it needs to be mentioned that the correlation of the anchors and MCS was lower than the correlation of the anchors and the PCS, which was also found in other studies [17, 44, 45]. Furthermore, the influence of dyspnea and physical activity measured via mMRC, BDI, NYHA, and 6MWD on HRQL was higher than the influence of clinical parameters as vital and diffusion capacity. Other studies also showed similar results with varying interpretation of the relevance of the correlation between pulmonary function and HRQL [16, 46–49].

Longitudinal analysis indicated sufficient psychometric properties, whereas the small number of observations limited the validity. Additionally, MIDs could not be estimated in all cases due to lacking sufficient correlation of anchors or missing normal distribution. If assumptions were given, the mean MIDs were higher compared to Swigris et al. (this study: range 5–21; Swigris et al.: range 2–4). Considering only the anchor with the highest correlation, the mean MIDs decreased and approached the MIDs of Swigris et al. Authors of the latter study used different methods and only two anchors [17]. Additionally, the amount of correlations or distribution patterns were not considered in providing MIDs. The different methods in combination with the strongly selected study sample of the BUILD-1 trial may explain the differences in our results.

The strength of this study lies in the international multicentre population of the IPF individuals of all ages and disease stages without strict inclusion and exclusion criteria, which provides a ‘real life’ setting and transferable results. We investigated a potential influence of the study sites and countries on HRQL. After adjusting for age, gender, DLCO % pred, FVC % pred and 6MWD there was no correlation with HRQL. The number of incorrect diagnoses should be negligible as the diagnosis was based on multidisciplinary discussion and on ATS/ERS/JRS/ALAT guideline criteria [4, 50]. To consider clinical and patient-centred values, we used objective anchors as lung function values (FVC % pred, DLCO % pred) and need of supplemental oxygen, (LTOT), as well as subjective parameters as dyspnea scores (self-reported by patients (mMRC, BDI/TDI) and physician (NYHA))and a measure of physical functioning (6MWD). The MID was estimated based on anchors as well as on distribution as widely recommended [51, 52].

Our study has several limitations. First of all, the follow-up intervals varied and only 62.6% of the study population had at least one follow-up SF-36. Additionally, in some cases the date of examination and visit was missing and the scheduled visit date was used as proxy instead. For example, in 19 of 364 analysed baseline and follow up SF-36 questionnaires the date needed to be approximated. The share of missing values of single items still met regulatory requirements. Some analyses were based on a small number of observations.

## Conclusion

SF-36 appears to be a valid instrument to measure HRQL in IPF and so can be used in RCTs or individual monitoring of this disease. Nevertheless, the additional evaluation of longitudinal aspects and MIDs can be recommended to further analyse these factors. Our findings have a great potential impact on the evaluation of IPF patients in clinical trials as well as individual disease monitoring.

## Additional files

Additional file 1: **Figure S1.** Missingness map: On the y axis the individuals are sorted based on the frequency of missing items. On the x axis there are the single items clustered by their dimension. Bright fields indicate missingness, dark fields indicate answered items. (DOCX 70 kb)

Additional file 2: **Figure S2.** Frequencies of answer categories on single item level including missing answers. The y axis shows the grouped items, the x axis indicates the frequency in numbers of the answer category or absence of answering, respectively. (DOCX 77 kb)

## Abbreviations

6MWD: 6 min walking distance
BDI: Baseline Dyspnoea Index
DLCO % pred: percent of predicted value of carbon monoxide diffusion capacity of the lung
FVC % pred: percent of the predicted value of forced vital capacity
GHP: general health perception
GLI: Global Lungs Initiative
LTOT: long-term oxygen therapy
MHI: mental health
mMRC: Modified Medical Research Council Dyspnea Scale
NYHA: modified New York Heart Association Classification
PAIN: bodily pain
PFI: physical functioning
ROLEM: emotional role functioning
ROLPH: physical role functioning
SOCIAL: social role functioning
TDI: Transitional Dyspnoea Index
VITAL: Vitality
