# Supplementary material for: Psychometric properties and minimal important differences of SF-36 in Idiopathic Pulmonary Fibrosis
Source: Respir Res. 2019 Mar 1;20:47. doi: 10.1186/s12931-019-1010-5 (PMC6397447; doi:10.1186/s12931-019-1010-5)
Supplement: Supplementary file 1 — Figure S1. Missingness map: On the y axis the individuals are sorted based on the frequency of missing items. On the x axis there are the single items clustered by their dimension. Bright fields indicate missingness, dark fields indicate answered items. (DOCX 70 kb) [file 12931_2019_1010_MOESM1_ESM.docx]

Appendix 1

Missingness map: On the y axis the individuals are sorted based on the frequency of missing items. On the x axis there are the single items clustered by their dimension. Bright fields indicate missingness, dark fields indicate answered items.

Abbreviations: GHP general health perceptions, MHI mental health, PAIN bodily pain, PFI physical functioning, ROLEM emotional role functioning, ROLPH physical role functioning, SOCIAL social role functioning, VITAL vitality, HCHANGE1 item indicating change in health status of the last years
