# Supplementary material for: Psychometric properties and minimal important differences of SF-36 in Idiopathic Pulmonary Fibrosis
Source: Respir Res. 2019 Mar 1;20:47. doi: 10.1186/s12931-019-1010-5 (PMC6397447; doi:10.1186/s12931-019-1010-5)
Supplement: Supplementary file 2 — Figure S2. Frequencies of answer categories on single item level including missing answers. The y axis shows the grouped items, the x axis indicates the frequency in numbers of the answer category or absence of answering, respectively. (DOCX 77 kb) [file 12931_2019_1010_MOESM2_ESM.docx]

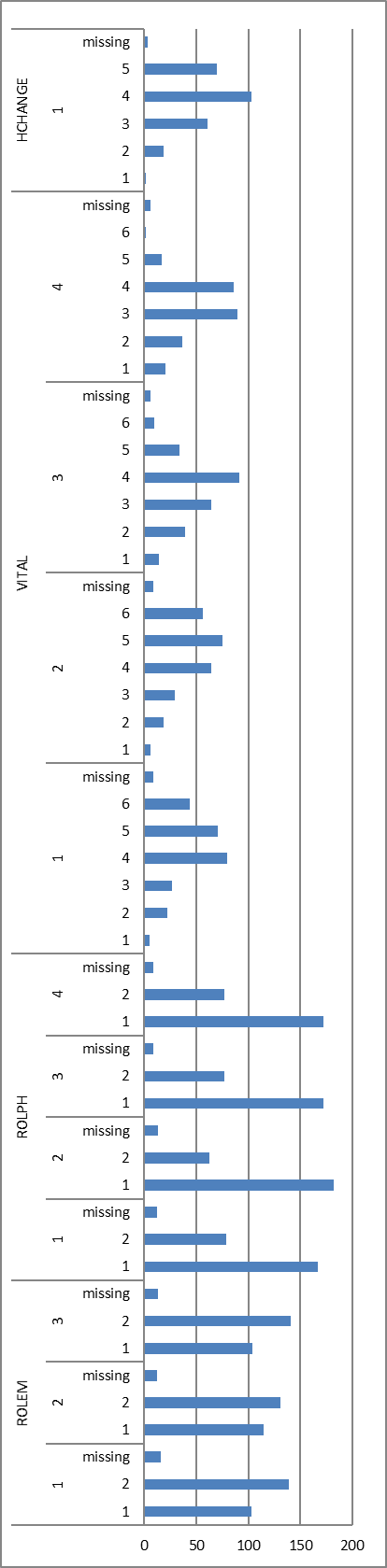

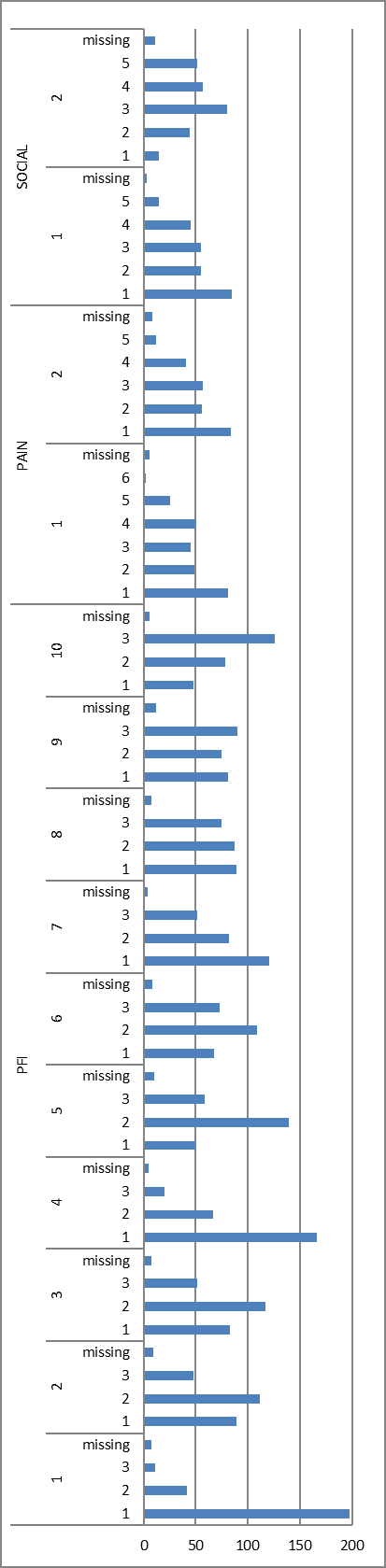

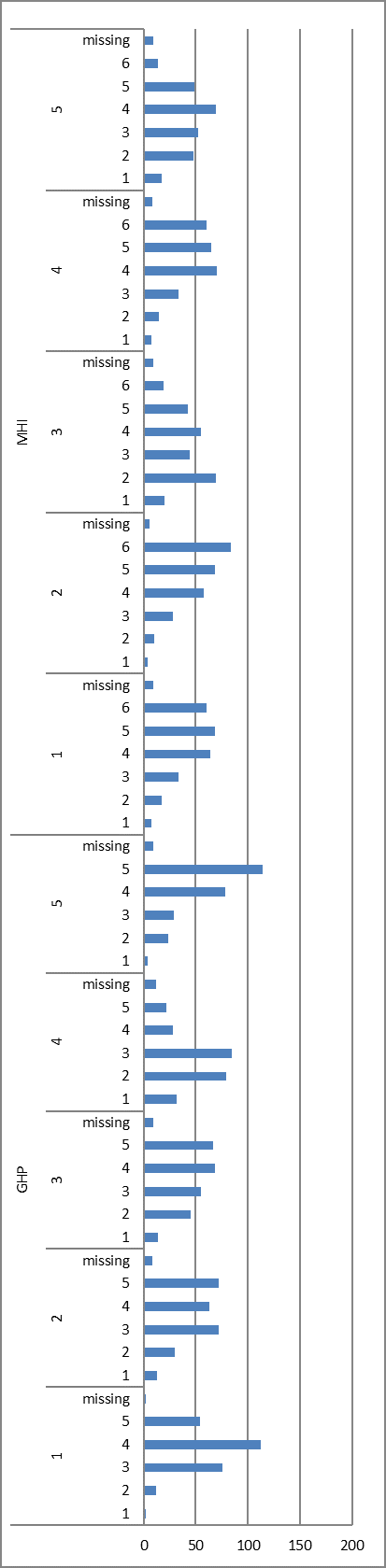


Appendix 2:

Frequencies of answer categories on single item level including missing answers. The y axis shows the grouped items, the x axis indicates the frequency in numbers of the answer category or absence of answering, respectively.

Abbreviations: GHP general health perceptions, MHI mental health, PAIN bodily pain, PFI physical functioning, ROLEM emotional role functioning, ROLPH physical role functioning, SOCIAL social role functioning, VITAL vitality, HCHANGE1 item indicating change in health status of the last years
